# Supplementary material for: Glucose Deprivation Induces Cancer Cell Death through Failure of ROS Regulation
Source: Int J Mol Sci. 2023 Jul 26;24(15):11969. doi: 10.3390/ijms241511969 (PMC10418724; doi:10.3390/ijms241511969)
Supplement: Supplementary file 1 [file ijms-24-11969-s001.zip › ijms-2516363-supplementary.pdf]

Supplementary Materials

1. Methods of the supplementary experiments

1.1. Seahorse Mito stress test assay

GSCs cells (1~2 × 10^4) were seeded in each well of a seahorse microplate and after 24 hours, cells were treated with or without glucose medium for 24 hours. To determine that ATP production was rescued by inhibiting ROS under glucose-free conditions, cells were pre-incubated with and without 5 mM NAC for 1 hour before changing to glucose-free medium. For oxygen consumption rate (OCR) determination, cells were incubated in XF base medium supplemented with 0 and 10 mM glucose, 1 mM sodium pyruvate, and 2 mM L-glutamine. Then cells were equilibrated in non-CO_2 incubator for 1 h before starting the assay. During the incubation, mitochondrial inhibitors oligomycin (1 μM), FCCP (0.5 μM), and rotenone/antimycin A (0.5 μM) dissolved in XF base medium were injected at the XFe96 sensor cartridge. Finally, normalization was performed with SRB assay.

1.2. FITC Annexin V and Propidium Iodide (PI) Cell Death Detection

Cell death was analyzed using annexin V-AbFlourTM 488 apoptosis detection kit (KTA0002, Abbkine scientific). Cells were cultured for 0, 12, and 24 h in glucose free condition. Cells werecollected, washed with cold PBS twice, centrifuged at 4°C for 3 min, and resuspended in 100 μL 1X annexin V binding buffer at a concentration of 2 × 10^5 cells/mL. The solution (100 μL)was added 4 μL of Annexin V-AbFlourTM 488 and 1 μL Propidium Iodide (PI) mix gently. The cells were incubated for 15 min at room temperature in the dark. 400 μL of 1X annexin V binding buffer was added to each tube, and the samples were analyzed by FACSVerse flowcytometry (BD Falcon, Bedford, MA, USA). Fluorescence intensity was quantified by FlowJo (Tree Star Inc., Ashland, OR, USA) software.

1.3. Lactate assay

Lactate levels were measured using the L-Lactate Assay Kit (ab65330, Abcam). 2 × 10^6 cells were washed with cold PBS, resuspened with lactate assay buffer and centrifuged at 4°C at top speed for 5 min. Collected supernatants were mixed with 50 μL of reaction reagents and incubated at room temperature for 30 min. The absorbance was measured at microreader OD 570 nm.

1.4. G6PDH assay

G6PDH activity was measured using the Glucose 6 Phosphate Dehydrogenase Assay Kit (ab102529, Abcan). Cells were washed with cold PBS and resuspended in 100 μl ice cold PBS (pH 6.5-8) quickly by pipetting a few times. Resuspended cells were kept on ice 10 min. Cells were centrifuged 4°C at 12,000 g for 5 min. Collected supernatants were mixed with 50 μL of reaction reagents and incubated at room temperature for 30 min. The absorbance was measured at microreader OD 450 nm.

1.5. ATP assay

ATP levels were measured using the ATP Assay Kit (ab83355, Abcam). Trypsinized cells were washed with cold PBS and lysed in ATP assay buffer and centrifuged at 4°C at top speed for 2 min.

Collected supernatants were mixed with 50 μL of reaction reagents and incubated at room temperature for 30 min. The absorbance was measured at microreader OD 570 nm.

1.6. GSH/GSSG ratio assay

GSH/GSSG ratio was performed using a EZ-Glutathione Assay Kit. Trypsinized cells were washed in cold PBS twice, suspended with cold 5% MPA, and homogenized by sonication. Samples were centrifuged at 4°C at 12,000 rpm for 10 min. To measure GSH, GSSG levels, collected supernatants were mixed with reaction reagents in 96-well plate separately. Then, the plate was incubated at room temperature 5 min and added 50 μl NADPH into each well. After the reaction, The absorbance was measured at microreader OD 412 nm.

2. Results of the supplementary experiments

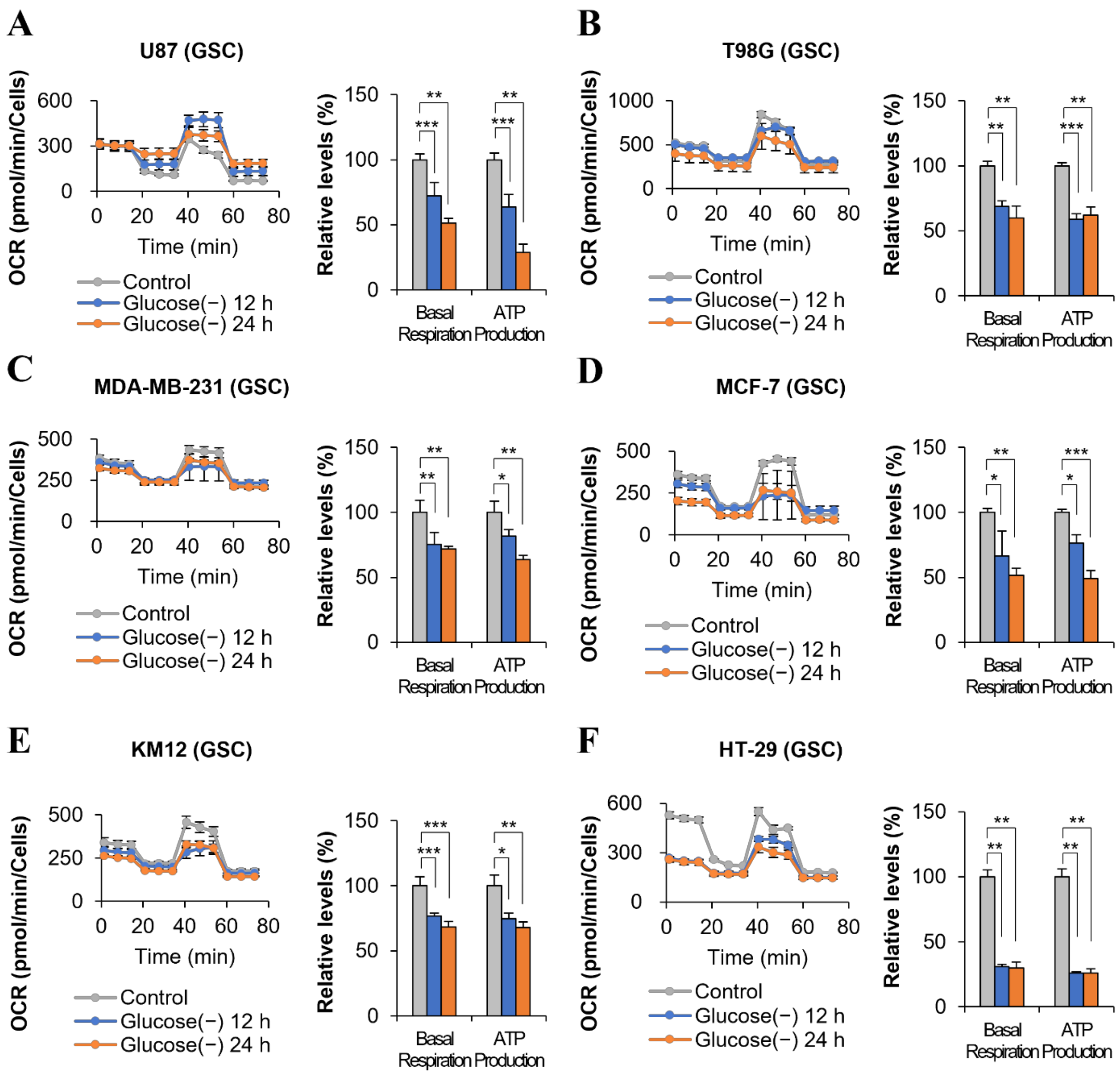

Figure S1. The other glucose-sensitive cancer cells are reduced ATP productions under the glucose deprivation.

(A-B) Basal respirations and ATP productions were measured by XFe96 extracellular flux analysis in Glioblastoma multiforme (GBM) cells (U87 and T98G) after glucose deprivation for 12 and 24 h (U87; n = 4, T98G; n = 4). (C-D) Basal respirations and ATP productions were measured by XFe96 extracellular flux analysis in breast cancer cells (MDA-MB-231 and MCF-7) after glucose deprivation for 12 and 24 h (MDA-MB-231; n = 4, MCF-7; n = 4). (E-F) Basal respirations and ATP productions were measured by XFe96 extracellular flux analysis in colon cancer cells (KM-12 and HT-29) after glucose deprivation for 0, 12, and 24 h (KM-12; n = 4, HT-29; n = 3). Error bars represent the mean + s.d. *, $p<0.05$; **, $p<0.01$; ***, $p<0.001$.

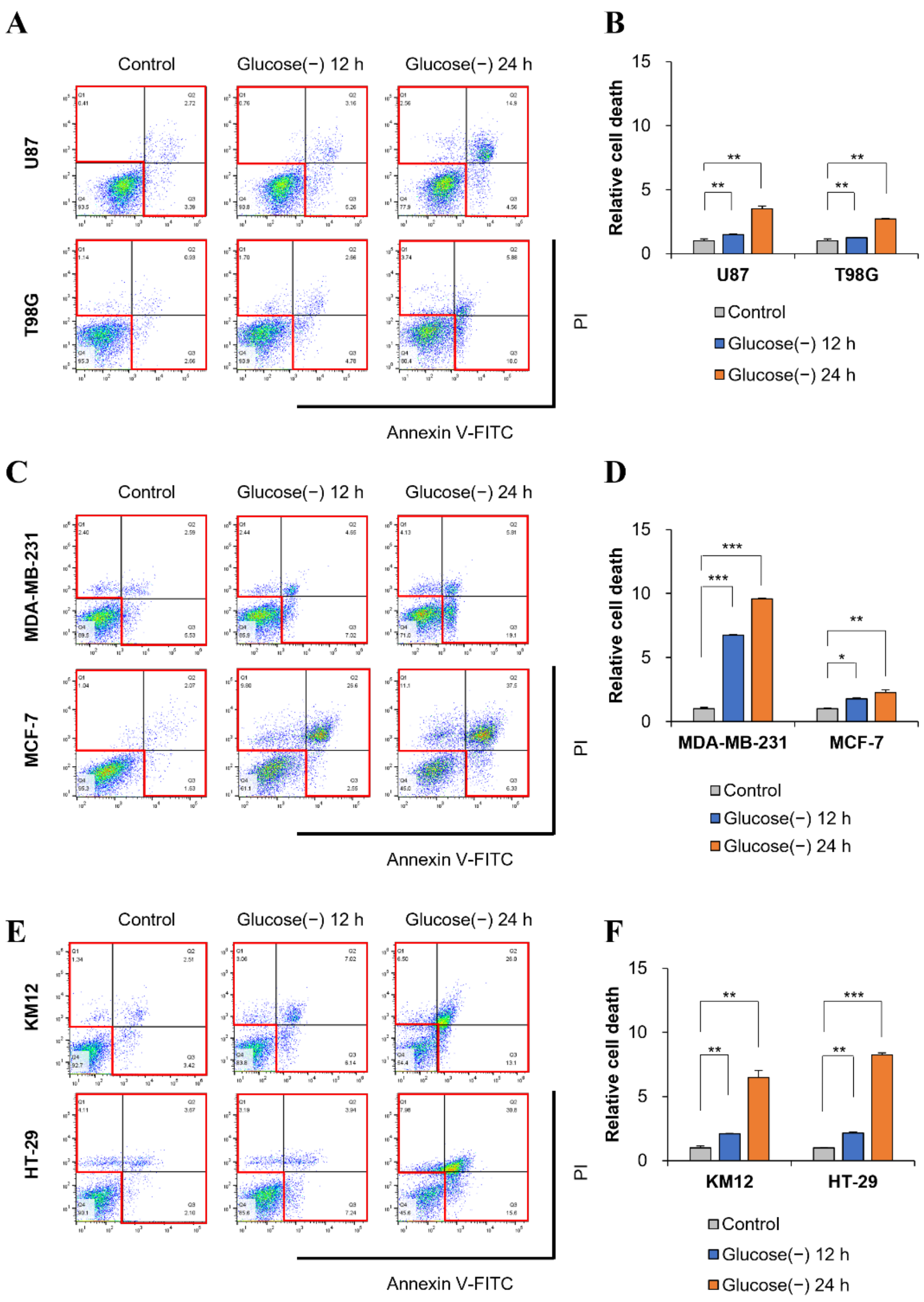

Figure S2. Glucose deprivation induces apoptosis in GSCs.

(A-B) Cell death was measured by annexin V and propidium iodide (PI) staining in Glioblastoma multiforme cells cultured under glucose deprivation conditions for 0, 12, and 24 h (n = 3). (C-D) Cell death was measured by annexin V and PI staining in breast cancer cells (GSCs) cultured under glucose deprivation conditions for 0, 12, and 24 h (n = 3). (E-F) Cell death was measured by annexin V and PI staining in colon cancer cells (GSCs) cultured under glucose deprivation conditions for 0, 12, and 24 h (n = 3). Apoptotic rates were quantified using FlowJo software. Error bars represent the mean + sd. *, $p < 0.05$; **, $p < 0.01$; ***, $p < 0.001$.

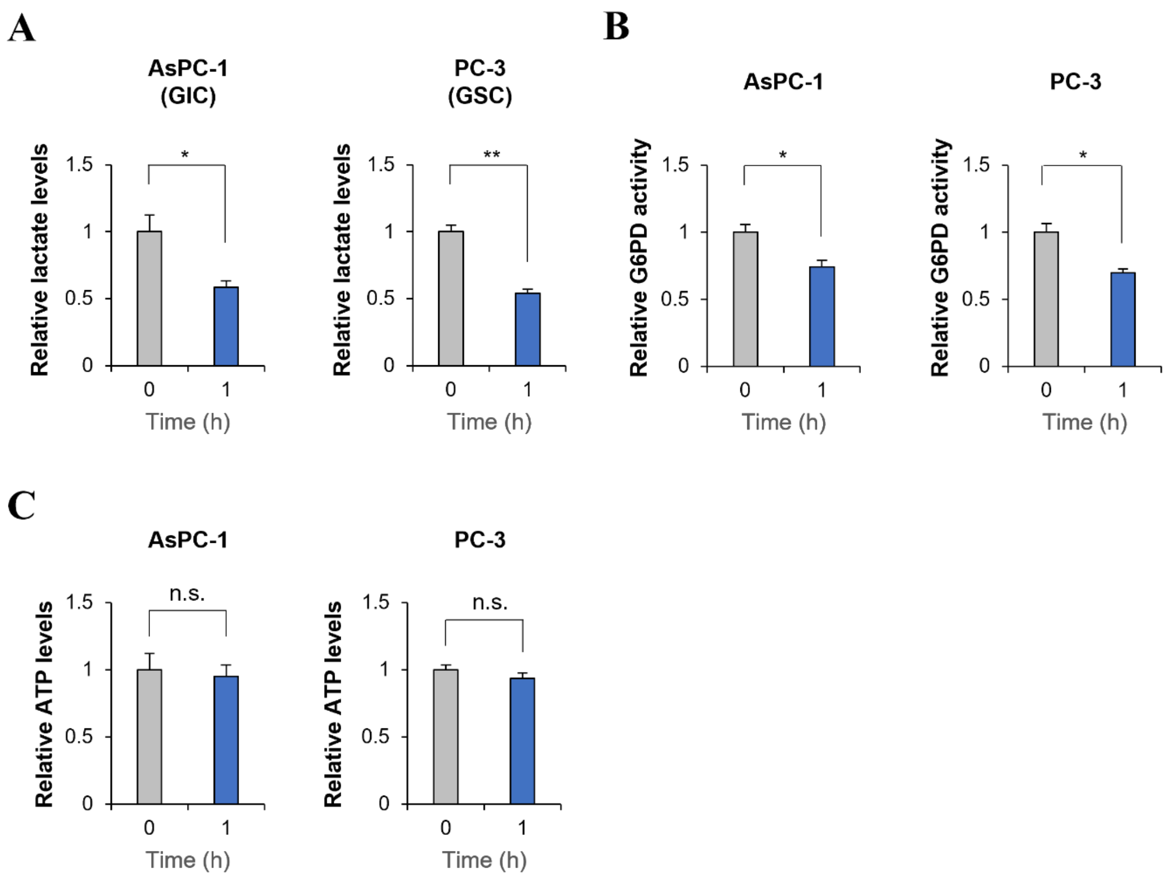

Figure S3. Even though lactate levels and G6PDH activity were decreased in short-term glucose deprivation, ATP levels were not changed.

(A) Lactate levels were measured after glucose deprivation for 0, 1 h (n = 3). (B) G6PDH activity was measured after glucose deprivation for 0, 1 h (n = 3). (C) ATP levels were measured after glucose deprivation for 0, 1h (n = 3). Error bars represent the mean + s.d. *, $p<0.05$; **, $p<0.01$; ***, $p<0.001$.

A

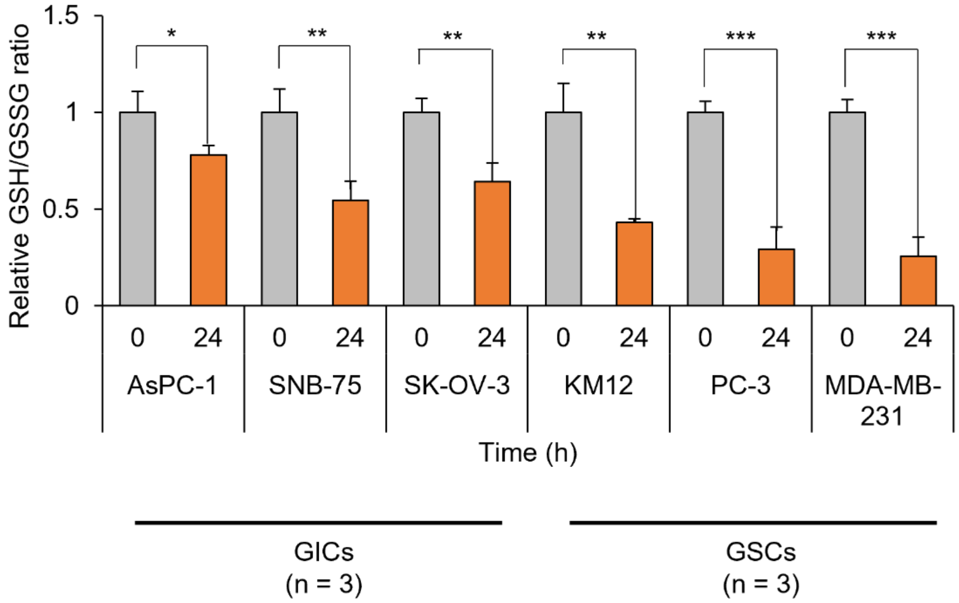

B

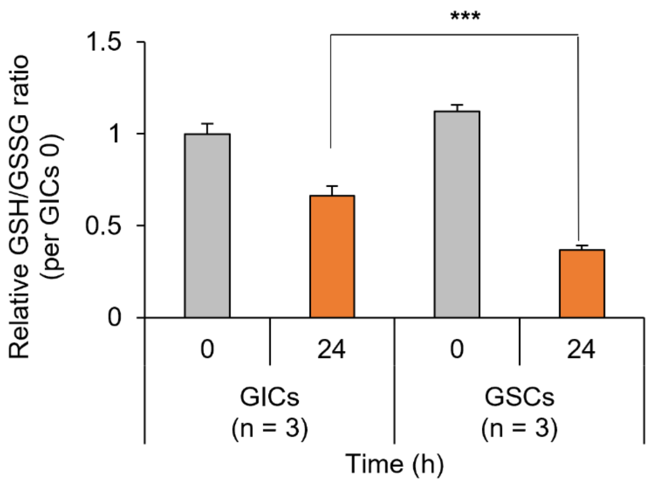

Figure S4. GSH/GSSG ratio levels of GIC was increased more than GSC under the glucose deprivation.

(A) GSH/GSSG ratio was measured using a Glutathione assay kit under the glucose deprivation for 0, and 24 h in GICs (AsPC-1, SNB-75, and SK-OV-3) and GSCs (KM12, PC-3, and MDA-MB-231) (n = 3). (B) The levels of relative GSH/GSSG ratio average were measured in GICs and GSC, respectively.

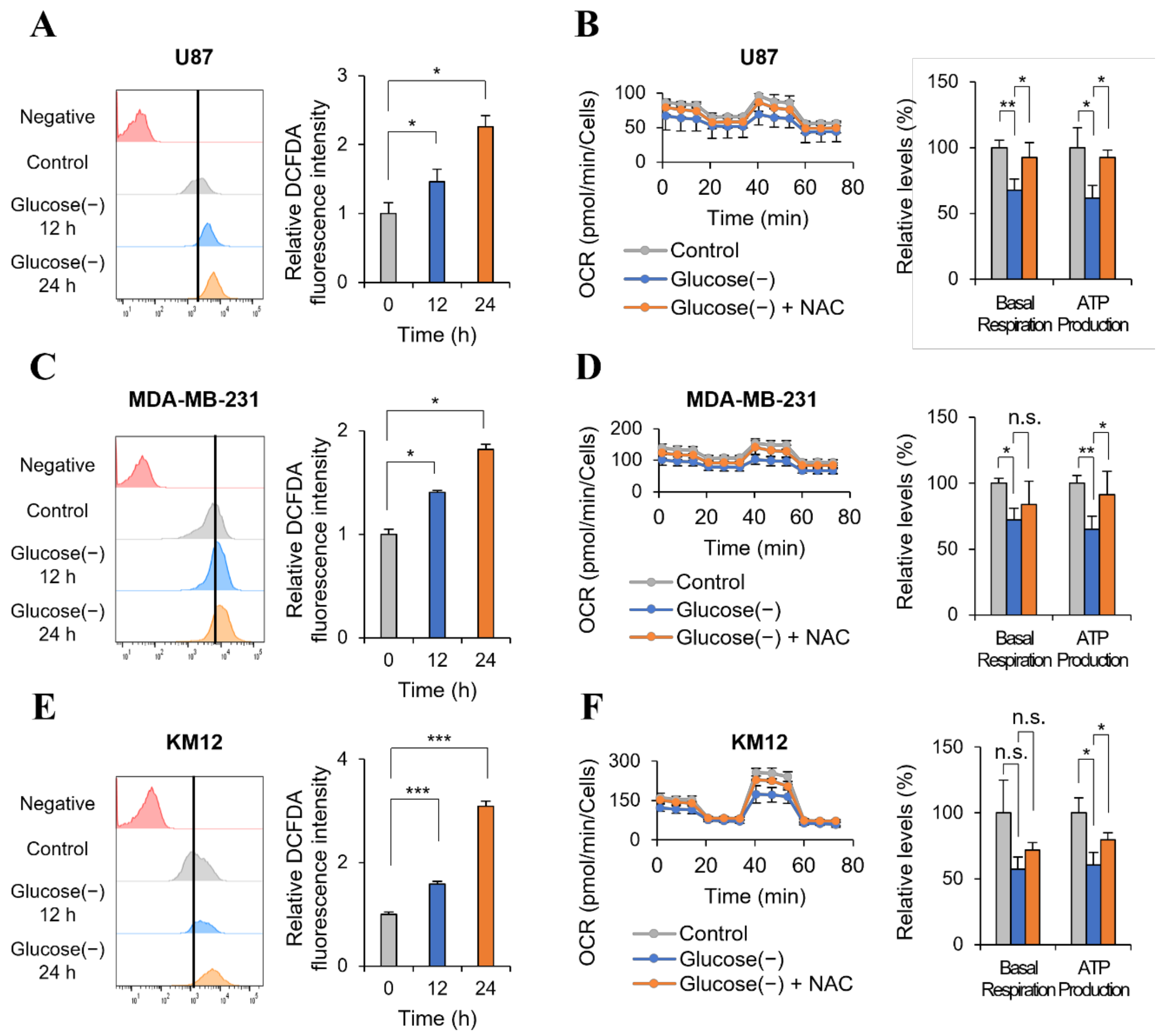

Figure S5. Glucose deprivation induces ROS and NAC treatment rescues ATP production by scavenging glucose deprivation-induced ROS in other GSC.

(A, C, E) ROS levels in glucose deprivation were analyzed by staining with DCFDA and determined by flow cytometry (n = 3). Fluorescence intensity was quantified by FlowJo software. (B, D, F) Basal respirations and ATP productions were measured by XFe96 extracellular flux analysis in GSC cells after glucose deprivation with or without NAC for 0 and 12 h (n = 3). Error bars represent the mean + s.d. *, $p<0.05$; **, $p<0.01$; ***, $p<0.001$.
